# Cervical Cancer Elimination Requires Systems, Trust, and Action

**DOI:** 10.3390/curroncol32100565

**Published:** 2025-10-08

**Authors:** Samara Perez

**Affiliations:** 1 Department of Oncology, Faculty of Medicine and Health Sciences, McGill University, Montréal, QC H4A 3T2, Canada; samara.perez@mcgill.ca; 2 Research Institute of McGill University Health Centre, Montréal, QC H3H 2R9, Canada; 3 Psychosocial Oncology Program, Division of Supportive & Palliative Care, Cedars Cancer Centre, McGill University Health Centre, Montréal, QC H4A 3J1, Canada

## 1. The Challenge Is Delivery

Cervical cancer has a clear and achievable path to elimination. Safe and effective HPV vaccines, sensitive HPV-based screening tests, self-collection methods, and established treatment protocols are already here. Fortunately, for cervical cancer, the science is no longer in question. What appears to stand in the way is the trust, the system, and implementing action. In Canada, for example, the decisive issue is whether the provincial governments will organize, fund, sustain, and maintain equitable programs that deliver these tools to everyone who needs them. Promises have been made, policy papers, and strategies drafted, but with each changing government we are left to ask: will Canada truly reach the goal of cervical cancer elimination by 2030?

## 2. Canada’s Uneven Progress

Canada has endorsed the World Health Organization’s 90–70–90 elimination targets: 90% of girls fully vaccinated by age 15, 70% of women screened by ages 35 and 45, and 90% of those with disease treated by 2030. Progress, however, is uneven and fragile. The National Advisory Committee on Immunization (NACI) now recommends a single-dose HPV vaccine schedule for immunocompetent individuals aged 9–20, simplifying logistics and reducing costs [1]. Yet, the implementation of routine school-based HPV vaccination remains uneven. Available provincial data reveal wide variation: some jurisdictions exceed 80% completion, while others fall below 50% [2,3]. Boys consistently lag behind girls, and Indigenous, rural, and newcomer youth are disproportionately under-vaccinated. The pandemic disruptions further widened these gaps. Confusion about Canada’s HPV vaccination rates also clouds the picture. For example, Han et al. (2025) report a weighted average of 86% for HPV first-dose coverage, drawing on the Childhood National Immunization Coverage Survey data [4,5]. But this data relied on only 1049 respondents, was self-reported, over-represented wealthier, urban households, and was conducted between December 2019 and April 2020, before the COVID-19 pandemic disrupted school-based programs. In contrast, our team in this Special Issue accessed provincial registries directly from the 2022–2024 school years, and estimated Canada’s true post-pandemic HPV vaccination completion rate closer to 64% [6]. It is dangerous to assume that Canada is within the 80–90% range when that is not borne out by current data. There may be pockets of high coverage, but this is not the national trend. Canada cannot truly measure a reliable and valid coverage of HPV vaccination uptake rates without a national vaccination registry.

Cervical screening shows similar inconsistencies. Leading the charge, British Columbia (BC) has fully implemented HPV primary testing, supported by a provincial registry and organized follow-up systems. Modeling from BC shows that the universal adoption of HPV testing could accelerate elimination by nearly a decade [7]. Prince Edward Island (PEI) has also completed its transition. A key element in PEI’s readiness was advocacy and a legislative change enabling nurses to follow up and refer directly to colposcopy. This shows how concrete government action, rather than policy promises alone. Therefore, PEI was able to remove barriers and make cervical screening systems work in practice in their province. Quebec has just begun the transition, and most other provinces continue to rely on Pap cytology despite its lower sensitivity. But again, without the system of organized screening programs, which is the case in Quebec, we will not be able to reach the WHO target of having 70% of women screened.

Self-collection is perhaps the most promising innovation. Studies demonstrate that it is as sensitive as clinician-collected sampling [8] and is highly acceptable across diverse groups [9]. For Canadians without access to primary care, which is nearly one-third in Quebec alone, self-collection removes the barrier of needing a clinician. Internationally, self-collection has transformed screening participation. As such, self-collection is an easy and feasible method for all females, not only for those who are under-screened or who live in remote areas. In Australia, real-world studies confirmed that self-collected samples were equivalent to clinician-collected samples. Based on this evidence, the National Cervical Screening Program expanded self-collection to the entire population in 2022, and by early 2025, 40% of HPV tests nationwide were self-collected [10]. With the exception of BC and PEI, self-collection remains confined to pilots and has not been systematically integrated into provincial health systems. Without investment, organized pathways, and sustained funding, its potential to reach under-screened populations will remain unrealized.

As noted in the opening remarks of this special issue, Canada’s progress toward cervical cancer elimination is real but fragile”. The barriers are not scientific. The challenges are systemic: the absence of national registries, uneven provincial rollouts, lack of or fragmented funding, and insufficient outreach to marginalized groups.

This Special Issue, Action and Impact: Prevention and Screening Strategies Contributing to the Elimination of Cervical Cancer, highlights how implementation science and knowledge translation can bridge these systemic gaps. Vahabi and colleagues (2024) conducted a qualitative study with sex workers and formerly incarcerated people, finding that self-collection was valued for its privacy, autonomy, and dignity, while conventional screening was often hindered by stigma, trauma, and mistrust [11]. Smith and colleagues tested a digital platform (*CervixCheck*) in BC, demonstrating that mailing and online access to self-screening kits in regions with historically lower uptake led to strong participation and follow-up [12]. Devotta and colleagues used concept mapping with South Asian women in Ontario and their providers, showing that, while providers prioritized education, women emphasized culturally safe communication, trust, and reducing stigma [13]. The special issues closes with a White Paper from an advisory committee consisting of Canadian from clinics, academia, research and community, with expertise in reducing cervical cancer incidence and advancing equitable healthcare led by Dr. Shannon Salvador, President, Society of Gynecologic Oncology of Canada. This article provides key clinical messages for both the public and government, alongside recommendations for HPV vaccination and screening. Together, these studies illustrate why KT and implementation research are critical for policymakers: they identify real-world barriers and solutions, guiding the design of equitable, scalable, and culturally safe strategies that lead to actual increase uptake of cervical screening. It is important for provincial leaders and health decision makers, e.g., Public Health Ontario, Ministère de la santé et des services sociaux to work alongside behavioural and implementations scientists and clinicians to develop systems and strategies to increase HPV vaccination and cervical screening

## 3. Global Lessons

Globally, 148 countries now offer HPV vaccination, yet only 15 have achieved 90% first-dose coverage. Weighted averages remain 61.6% for first dose and 47.6% for full coverage among girls aged 9–14 [4]. Rwanda and Uganda outperform many high-income nations by achieving more than 90% uptake through school-based and community-driven programs. In contrast, middle-income countries often falter because of systemic barriers and/or attitudes and beliefs.

Canada must look to Australia which shows what coordinated systems can deliver. By adopting national HPV primary testing, making self-collection universally available, and maintaining a centralized registry, Australia is projected to eliminate cervical cancer within a decade. Rwanda, with limited resources, has achieved extraordinary vaccine coverage by mobilizing schools, trusted community leaders, and local health workers and using implementation science frameworks [14,15]. These examples underscore that elimination is not driven by scientific discovery but by infrastructure, trust, and political commitment.

## 4. Beyond Science: The Human Who Suffers from Cervical Cancer

The psychosocial barriers are equally significant. Cervical cancer continues to be a “lonely cancer,” tied to sexuality and reproductive identity, and too often marked by stigma, shame, and silence. Cervical cancer causes ~350,000 deaths worldwide, among which there are 400 Canadians. These are lives that can be saved. Building trust requires engagement beyond the clinic. Organizations such as HPV Global Action, which has reached over 100,000 students across Canada through school-based education and awareness campaigns, demonstrate how community-led efforts can shift attitudes and normalize prevention [3]. Psychosocial oncology research adds that trust, autonomy, and feeling heard are central to engagement. Framing HPV prevention as cancer prevention for all genders, rather than solely as sexual health, is critical to broad acceptance. With respect to HPV vaccine hesitancy, the issues are not unique to HPV, and is shaped by general attitudes and barriers towards vaccinations. Indigenous, racialized, and newcomer communities continue to face systemic inequities that erode trust in health systems [16].

## 5. What Canada Must Do

If Canada is to meet its elimination target, governments and decision makers must act with urgency and decisively. Vaccination programs must be harmonized across provinces and supported with consistent funding. As the COVID-19 pandemic showed, Canada can mobilize coordinated immunization strategies, and it must now do so again. A “once eligible, always eligible” policy should extend publicly funded vaccination to adults up to age 45 [17]. National vaccination and screening registries must be established to monitor progress and identify equity gaps. HPV primary testing must be rolled out nationwide, with universal access to self-collection and robust follow-up pathways. Above all, governments must invest in culturally safe, community-driven outreach that builds trust and ensures that no one is left behind.

## 6. Conclusions

Cervical cancer can be the first cancer eliminated entirely from our world. But this will not happen through science alone. Canada has the evidence and the tools. What is missing is political urgency, system-wide coordination, and the trust and mobilization of the people these programs are meant to serve. The science has already done its part. With the support of the public, the responsibility now lies with governments and systems to roll up their sleeves and make HPV vaccination and screening real, implemented, and sustained, not just promised on paper or in the press, but delivered equitably in practice. Cervical cancer can be eliminated—let us work together to make it happen by 2030.
